# Rapid response to the COVID-19 pandemic: Vietnam government’s experience and preliminary success

**DOI:** 10.7189/jogh.10.020502

**Published:** 2020-12

**Authors:** Thi Phuong Thao Tran, Thanh Ha Le, Thi Ngoc Phuong Nguyen, Van Minh Hoang

**Affiliations:** 1 Hanoi University of Public Health, Hanoi, Vietnam; 2 Graduate School of Cancer Science and Policy, National Cancer Center, Goyang-si, Republic of Korea

## Abstract

**Background:**

The COVID-19 pandemic has hit all corners of the world, challenging governments to act promptly in controlling the spread of the pandemic. Due to limited resources and inferior technological capacities, developing countries including Vietnam have faced many challenges in combating the pandemic. Since the first cases were detected on 23 January 2020, Vietnam has undergone a 3-month fierce battle to control the outbreak with stringent measures from the government to mitigate the adverse impacts. In this study, we aim to give insights into the Vietnamese government’s progress during the first three months of the outbreak. Additionally, we relatively compare Vietnam’s response with that of other Southeast Asia countries to deliver a clear and comprehensive view on disease control strategies.

**Methods:**

The data on the number of COVID-19 confirmed and recovered cases in Vietnam was obtained from the Dashboard for COVID-19 statistics of the Ministry of Health (https://ncov.vncdc.gov.vn/). The review on Vietnam’s country-level responses was conducted by searching for relevant government documents issued on the online database ‘Vietnam Laws Repository’ (https://thuvienphapluat.vn/en/index.aspx), with the grey literature on Google and relevant official websites. A stringency index of government policies and the countries’ respective numbers of confirmed cases of nine Southeast Asian countries were adapted from the Oxford COVID-19 Government Response Tracker (https://www.bsg.ox.ac.uk/research/research-projects/coronavirus-government-response-tracker). All data was updated as of 24 April 2020.

**Results:**

Preliminary positive results have been achieved given that the nation confirmed no new community-transmitted cases since 16 April and zero COVID-19 – related deaths throughout the 3-month pandemic period. To date, the pandemic has been successfully controlled thanks to the Vietnamese government’s prompt, proactive and decisive responses including mobilization of the health care systems, security forces, economic policies, along with a creative and effective communication campaign corresponding with crucial milestones of the epidemic’s progression.

**Conclusions:**

Vietnam could be one of the role models in pandemic control for low-resource settings. As the pandemic is still ongoing in an unpredictable trajectory, disease control measures should continue to be put in place in the foreseeable short term.

The COVID-19 outbreak which originated from Wuhan city, China has widely expanded to 211 countries and had been declared as a public health emergency by the World Health Organization. As of 24 April, the total number of confirmed cases and deaths with COVID-19 globally was 2.626.321 and 181.938, respectively [1]. The burden of the pandemic varies across countries. The case-facility rate was high in several countries including the United States (5.6%), Spain (10.4%), Italy (13.4%), and the UK (13.6%) [2]. This pandemic is much more than a health crisis that seriously disrupts human lives and economic activities all over the world.

When compared to other regions, Southeast Asia also has experienced a significant rise in cases, notably in countries like Singapore, Indonesia, Philippines and Malaysia [3]. The crisis has challenged governments to respond urgently in the same manner they would have done had it been a war or natural disaster. Countries are making every effort to slow the spread of the virus through testing and treating patients, tracing contacts, limiting travels, quarantining citizens and so forth. Countries’ timely responses play a crucial role in controlling the spread of the pandemic.

The first detected cases on 23 January has marked the beginning of Vietnam’s 3-month fierce battle against COVID-19 with complicated and unpredictable progression of the epidemic. As most countries, Vietnam has also been suffering and will likely continue to shoulder the devastating impacts on health, economy and society over the months and years to come. In response to the pandemic, considerable efforts of the government have been made such as enforcing border closure, entry ban, isolation and quarantine; improving health care capacity; executing information campaigns amongst others to reduce the adverse impacts of the pandemic. This study aims to bring forth a profound overview of how the Vietnamese government prepared and responded in the wake of COVID-19, during the first three months of the pandemic. Additionally, we relatively compare Vietnam’s response with that of other Southeast Asia countries to deliver a clear and comprehensive view on disease control strategies. Our research could potentially contribute to the literature on coping strategies in times of unprecedented pandemics and serve as reference to help countries prepare for similar future outbreaks.

## METHODS

### Data sources

The data on the number of COVID-19 confirmed and recovered cases in Vietnam was obtained from the Dashboard for COVID-19 statistics of the Ministry of Health (MOH) (https://ncov.vncdc.gov.vn/) as of 24 April 2020 [4].

The review on Vietnam’s country-level responses was conducted by searching for relevant documents issued by the government on the online database ‘Vietnam Laws Repository’ (https://thuvienphapluat.vn/en/index.aspx) [5]. Search terms were ‘coronavirus’ or ‘COVID-19’. In addition, grey literature on Google and relevant official websites were searched. Inclusion criteria was documents focusing on national-level regulations in response to the COVID-19 pandemic that were published up until 24 April 2020.

For the cross-comparison of policies among Southeast Asian countries (including Brunei, Laos, Indonesia, Malaysia, Myanmar, Philippines, Singapore, Thailand and Vietnam), a stringency index of government policies and the countries’ respective numbers of confirmed cases were adapted from the Oxford COVID-19 Government Response Tracker (https://www.bsg.ox.ac.uk/research/research-projects/coronavirus-government-response-tracker) [6]. The stringency index was calculated based on nine indicators of government’s responses, including school closing, workplace closing, cancellation of public events, restriction on gathering size, public transport closure, “shelter-in-place” and home confinement orders, restriction on domestic/internal movement, restriction on international travel and public information campaign. The stringency index ranged from 0 to 100 in which the higher the score, the stricter the government policies. The stringency index is not intended to measure the appropriateness or effectiveness of a country’s response but rather to provide a stringency indicator of the governments’ policies. The data was updated as of 24 April 2020.

### Data management and analysis

**Figure 1:**
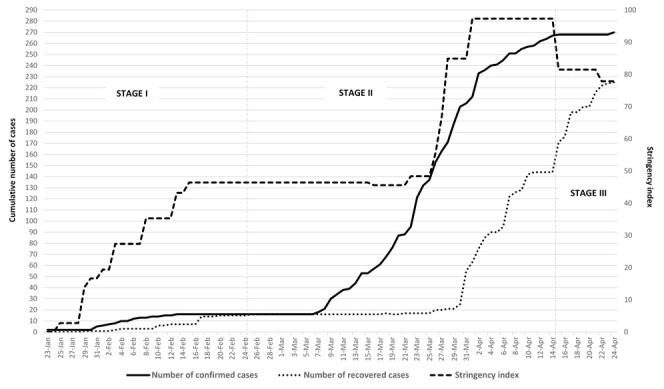
The cumulative number of confirmed cases and recovered cases with COVID-19, by stages.

The daily cumulative number of confirmed cases and recovered cases of COVID-19 in Vietnam from 23 January to 24 April were entered into Excel (Microsoft Inc, Seattle, WA, USA). We downloaded the Excel file comprising of the stringency index and the number of confirmed cases for all Southeast Asian countries from the Oxford COVID-19 Government Response Tracker. In order to provide an overview of the COVID-19 pandemic in Vietnam, changes in the cumulative number of confirmed cases, number of recovered cases and stringency index during the 3-month period are illustrated in **Figure 1**. Additionally, fluctuations of stringency indexes of each Southeast Asian country by increasing confirmed cases were presented to compare policy responses among these nations. All figures were drawn through Excel.

## RESULTS AND DISCUSSION

### Overview of the COVID-19 pandemic in Vietnam

The first 3-month progress of the COVID-19 outbreak in Vietnam can be temporarily divided into three stages (**Figure 1**). Stage I was from 23 January (the first case detected) to 25 February, during which the number of confirmed cases with COVID-19 increased slowly to 16 on 25 February. Most of the cases in this stage were people with recorded travels from China. In Stage II, the new cases were initially those identified with traveling histories to South Korea and European countries, but later served as the sources of community transmission, ramping up the total number of confirmed cases to 268 on 15 April. In Stage III, only 2 new cases were reported, 225 out of 270 cases (83.3%) had recovered as of 24 April. There were no deaths recorded as of 24 April 2020.

Vietnam has gained preliminary promising achievements in controlling the COVID-19 pandemic given that the nation has maintained a clean record of new COVID-19 cases for eight days since 16 April and has had zero deaths for three months straight. These are attributable to the rapid, strong and decisive efforts of the government in the wake of the outbreak. This is a commendable accomplishment, given that Vietnam shares a long and bustling border with China – the initial hotspot of the outbreak. However, Vietnam may confront further challenges in the upcoming months due to the vast flows of Vietnamese citizens returning home from abroad and the number of patients who are tested positive again after being discharged from hospital.

### Vietnamese government’s response to the COVID-19 pandemic

In hindsight, the epidemic is still relatively under control in Vietnam thanks to the continued prompt and proactive precautions taken by the government. The stringency index, which simply reflects the strictness in counter-measures, has changed over time to correspond with crucial progression milestones during the three stages of the pandemic. In stage I which marks the initial period of the outbreak, the government took a step-by-step operationalization of intensive regulations to combat the outbreak and this approach continued to be reinforced during the first haft of stage II. Rapid community transmissions of the virus in the second haft of stage II prompted the government to put in place the strictest counter-measures, resulting in the 15-day national social distancing with the highest level of stringency (scored nearly 100 in stringency index). In the ongoing stage III as of 24 April, the epidemic has been relatively controlled, enabling social distancing measure relieves (**Table 1**).

**Table 1:** Summary of Vietnamese government’s response to COVID-19 outbreak

Indicator	Policies/Regulations
Border closure and entry ban	**Stage I:**
	• On 23 January, The Civil Aviation Authority of Vietnam suspended all flights to Wuhan – where the initial outbreak of nCoV was reported. Instruction No. 358/CT-CHK [7].
	• On 29 January, the Civil Aviation Authority of Vietnam temporarily halted the granting of permission for new flights from Vietnam to areas in China that have been affected by the epidemic and ceased all flights from those areas to Vietnam [8].
	• On 30 January, Vietnam suspended visa issuance to Chinese tourists as a temporary measure. Vietnam also temporarily refused entry for all foreign visitors who had been to mainland China (including transit) in the 14 d prior to their intended arrival in Vietnam [5].
	• On 1 February, the Civil Aviation Authority of Vietnam suspended all flights to mainland China, Hong Kong, Macau, and Taiwan. However, the ban on flights to Hong Kong, Macau, and Taiwan was lifted from 2 February. Meanwhile, all flights to and from mainland China (including transits) were still suspended [8].
	• On 12 February, Vietnam and China agreed to reopen border crossings to ease congestion and normalize trade (Telegraph No. 224/CD-TTg dated 12 February 2020). Several border crossings in Quang Ninh, Lai Chau, Lao Cai, Lang Son, and Ha Giang were reopened. However, backlogs remained and other border crossings remained closed [9].
	**Stage II and III:**
	• On 25 February, Vietnam temporarily imposed entry bans on people with recent travel records from South Korea and the epidemic-affected areas [10].
	• On 29 February, Vietnam suspended the Visa Waiver Program for South Korean nationals. In addition, all incoming travelers from South Korea were required to complete a mandatory 14-d quarantine [11].
	• On 5 March, Vietnam Airlines and low-cost carrier Vietjet Air temporarily halted all flights to and from South Korea [12].
	• On 18 March, Vietnam Airlines temporarily suspended flights between Vietnam and Malaysia until the end of March due to the Malaysian government imposing border closure during this time. Additionally, flights between Vietnam and France were indefinitely suspended. Moreover, foreigners who entered or transited through ASEAN countries within the past 14 d were required by the Vietnamese government to undergo a 14-d mandatory quarantine upon arrival in Vietnam [13].
	• On 19 March, Vietnam Airlines temporarily suspended international flights [14].
	• On 29 March, the Ministry of Transport decided to reduce the number of domestic flights between 30th March and 15th April 2020. More specifically, all domestic routes were temporarily suspended during the above period, except for routes between Hanoi and Ho Chi Minh city and between Hanoi / HCM City and Da Nang / Phu Quoc [10].
	• On 1 April, border crossings between Vietnam and Cambodia and Laos were temporarily closed during the period from 1 April to 15 April [15].
Quarantine and lockdown	**Stage I:**
	• On 2 February, the Official Dispatch No. 156/CD-TTg implementation of a 14-d quarantine for Vietnamese citizens returning from China was signed by Prime Minister Nguyen Xuan Phuc [16].
	• On 3 February, the Prime Minister ordered all travelers who came from or transited through 31 provinces of China within the last 14 d to be quarantined [17].
	**Stage II:**
	• From 26 February, Vietnamese passengers who traveled from the Republic of Korea would be quarantined for 14 d after entering Vietnam. Besides, Vietnam would reject foreigners who have visited or transited through Daegu and Gyeongsangbuk of South Korea, except for those entering Vietnam for business purposes (14 d quarantine is compulsory) [18].
	• On 1 March, Hanoi and Ho Chi Minh city airports stopped receiving all flights from South Korea, instead they were rerouted to land at Van Don (Quang Ninh province) and Can Tho airports. Before entering Vietnam, all passengers on these flights had to undergo mandatory health checks and isolation for 14 d at specific locations arranged by Vietnamese regulators. All passengers who booked flights from or transited through Italy or Iran within 14 d prior to landing in Vietnam would also be isolated for 14 d [19].
	• From 1 March, all people who entered the country from epidemic countries (China, Korea, Italy, Iran) must fill out a medical declaration upon arrival. In the case of passengers showing signs of common COVID-19 symptoms (fever, cough, or shortness of breath), they would be transferred to designated health facilities for quarantine, treatment and sample collections of clinical specimens for COVID-19 testing [20].
	• On 7 March, the Health Ministry required all arrivals from March 1 to self-isolate at home if they had not been quarantined in a centralized zone in the past 14 d [21].
	• On 21 March, Vietnam announced a mandatory quarantine for all passengers from all countries and regions upon entry to Vietnam. For passengers holding diplomatic or official passports, if he/she was in normal health condition, had no signs of illness and was guaranteed by the embassies or representative offices who ensured the quarantine conditions, he/she may undergo quarantine at the representative office or place of residence for 14 d from the arrival date [22].
	• On 1 April, Vietnam government enacted the Directive No. 16/CT-TTg on implementing strict social distancing rules nationwide for 15 d to prevent the spread of COVID-19. The measures included self-isolation and people were only allowed to leave their homes for food and medicines. Gathering of more than two people was also banned, together with a must two-meter distance rule between people in public areas. Factories, businesses and service establishments producing and providing essential goods were allowed to remain open but must follow strict health guidelines [23].
	• On 13 April, authorities ordered people working at a unit of Samsung Display in Bac Ninh province to be quarantined and the Samsung factory was isolated after a worker was tested positive for COVID-19 [24].
	• Certain localities continued to be locked down to curb the spread of COVID-19, such as Son Loi (Vinh Phuc), Truc Bach (Hanoi), Viet Hai and Gia Luan (Hai Phong), Ha Loi (Me Linh, Hanoi), Bach Mai hospital (Hanoi) [25-29].
	**Stage III:**
	• On 16 April, Vietnam divided all localities into ‘high-risk, ‘at-risk’, and ‘low-risk’ zones to implement social distancing measures related to the pandemic. Vietnam extended the social distancing measures at least until 22 April for high-risk and at-risk localities, which included Hanoi, HCM City, as well as ten other provinces. Depending on the situation in high-risk areas, the measures could be extended to 30 April [30].
	• From 23 April, Vietnam loosened the social distancing measures, though the restrictions continued in some high-risk areas. While social distancing in Hanoi and HCM city was largely lifted, some restrictions continued to remain for restaurants, bars, clubs [31].
Non-essential business, school/workplace, public transport closure	**Stage I:**
	• On 2 February, the Department of Education and Training of Ho Chi Minh City and Hanoi issued for closure of all pre-school levels, elementary schools, high schools and all regular education institutions [32,33].
	• On 3 February, Prime Minister Nguyen Xuan Phuc signed the Official Dispatch No. 156/CD-TTg on strengthening the prevention and control of the coronavirus outbreak, which included restrictions on large gatherings and festivals including the inaugural ones [34].
	• On 3 February, Prime Minister requested the Ministry of Education and Training to provide specific guidance for students studying at home (in Official Dispatch No. 156/CD-TTg). By the end of 8th February 63/63 provinces and cities that had extended the closure of schools in their areas continued to extend this until 22 April [35].
	• On 3 February, the Official Telegraph No. 396/CD-BVHTTDL, Ministry of Culture, Sports, and Tourism required to prolong temporary suspension of festivals and activities at historical monuments and sites [36].
	**Stage II:**
	• On 16 March, Vietnam railway authorities suspended several domestic routes from Hanoi and HCM City [37].
	• On 24 March, the People's Committee of HCM city issued a decision to close all non-essential services like beauty clinics, karaoke, massage parlors, bars, entertainment venues in 24 districts, effective immediately [38].
	• On 27 March, the government asked religious organizations to cancel religious festivals, entertainment activities and conferences that attract large crowds as a precaution; to suspend all non-essential business; ban on gathering of more than 10 people in outside areas [39].
	• On 27 March, Hanoi stopped public transport activities by 80%. After one day, all bus operations were halted in response to COVID-19 from 28 March to 15 April. This rule was extended for another week until 22 April [40].
	• Vietnam banned gatherings of more than 20 people for at least two weeks starting from March 28 and temporarily shut down services like massage parlors, tourist sites and cinemas nationwide. In addition, major cities like HCM City, Hanoi, Can Tho and Da Nang needed to temporarily shut down all service facilities except for food, pharmacy and medical treatment services [39].
	• On 30 March, the Ministry of Transport issued Official Dispatch No. 2917/BGTVT-VT on stopping the operation of contract vehicles had 9 seats and above coming to or departing from Hanoi, HCM City [41].
	• On 1 April, Hanoi, HCM City and Da Nang requested to stop all construction activities from 1 April to 15 April [23].
	• Until 1 April, in line with the national quarantine, domestic flights were significantly reduced to only one round trip a day between Hanoi and HCM city; Hanoi and Da Nang; HCM City and Da Nang. Passenger trains between Hanoi and HCM City were also limited to two trips per day. Public transport services were suspended while transport from region to region were minimized except for essential services [42].
	• Also on the same day, the Ministry of Transport issued the Official Dispatch No. 3064/BGTVT-VT to prohibit the transportation activities of domestic flights and inter-provincial transport routes, as well as the transportation activities of contract vehicles, tourist cars, taxis, buses in all provinces and cities [43,44].
	**Stage III:**
	• On 16 April, the Minister of Transport issued the Official Dispatch No. 3655/BGTVT-VT: Adjusting the transportation plan accordingly in the three area groups at risk of COVID-19 (I high-risk, II at-risk and III low-risk). Specifically, with provinces in Group I and Group II, no inter-provincial passenger transportation would be carried out until 22 April. Vietnamese air passenger carriers were expected to resume regular domestic flights but stated that routes and flight frequency might be subject to change [45].
	• On 23 April, the social distancing campaign was stopped in 28 cities and provinces and the Prime Minister implemented more relaxed restrictions than before: Both Hanoi and HCM City (now classified only as “at risk”), as well as 10 other localities would still have to stop socializing activities, which include all events of over 20 people and gatherings of 10 people or more outside workplaces, schools and hospitals. However, shops, street businesses and non-essential services were allowed to reopen [30].
	• On 23 April, the frequency of domestic flights on the Hanoi - HCM city route was increased, as well as reopening of other domestic routes with limitation [46,47].
	• From 23 April, several schools were reopened in some provinces [48].
Improving capacity of health care systems	• On 16 January, MOH issued Decision No. 125/QD-BYT on the first guideline for diagnosis and treatment of coronavirus infection [49]. The guideline was updated continuously in Decision No. 322/QD-BYT on 06 February, in Decision No. 1344/QD-BYT on 25 March [50,51].
	• On 28 January, Prime Minister Nguyen Xuan Phuc signed the Directive No. 05/CT-TTg on the prevention and control of the Coronavirus outbreak, which immediately requested to establish a Rapid Response Team and also requested MOH to report daily the situation to the Prime Minister [52].
	• On 30 January, MOH issued Decision No. 255/QĐ+D-BYT on the establishment of Rapid Response Teams [53].
	• On 30 January, the Prime Minister issued the Decision No. 170/QD-TTg on establishment of National Steering Committee for controlling the outbreak [54].
	• On 1 February, MOH launched the hotlines 19009095 and 19003228 for the prevention and control of the coronavirus outbreak [55]. On 22 March, all provinces/cities operated their own hotlines for counseling [56].
	• On 7 February, the National Institute of Hygiene and Epidemiology successfully cultured and isolated the new coronavirus in the laboratory [57].
	• On 7 February, the National Steering Committee for prevention and control of the coronavirus outbreak issued Decision No. 80/QD-BCDQG on establishment of Sub-committees to respond to the outbreak [58].
	• On 5 March, the Agency of Health Examination and Treatment launched the online management and administration system for diagnosis and treatment of COVID-19 infection [59].
	• On 5 March, Vietnam successfully produced the virus detection test kit (RT-PCR and real-time RT-PCR) [60].
	• On 30 March, MOH announced imports of 200 000 rapid test nCoV from South Korea. These test kit were prioritized for people who self-isolated at home, in concentrated isolation areas and in high-risk areas [61].
	• On 3 April, the Prime Minister required MOH to speed up the production of medical equipment with high quality and reasonable prices [62].
	• On 16 April, the Drug Administration of Vietnam issued Official Dispatch No. 4162/QLD-KD stopping anti-COVID-19 drugs exports [63].
	• On 18 April, MOH launched an online-based medical examination and treatment system to support hospitals in remote areas regarding counseling, consultation, imaging diagnosis, pathology..[64].
	• In addition, MOH issued numerous guidelines for quarantine at home, at quarantine zones, at residential areas for the prevention and control of the COVID-19 spread [65-67]; Guidance on prevention and management of COVID-19 infection for pregnant women and infants, for the elderly and people with chronic diseases [68,69]; Recommendations on prevention and control of the COVID-19 at apartment buildings, at workplaces, at shopping malls, restaurants…[70-72].
Information campaign	• On 30 January, MOH issued Official Dispatch No.369/BYT-TT-KT on propaganda strengthening, advocacy of prevention and control of nCoV to the People's Committee of 63 provinces [73].
	• On 2 February, the Ministry of Information and Communications issued Directive No. 05/CT-BTTTT on the implementation of prevention and control of the new coronavirus outbreak [74], in which: - Providing information through text messaging for each mobile subscriber. - Producing videos, short films to disseminate anti-nCoV information on social networks such as Facebook, Zalo, Youtube, Lotus. - Launching the hashtag #ICT_anti_nCoV to raise awareness. - Disseminating information through the media such as newspaper, radio, television. - Ensuring and improving collaboration and communication amongst hospitals and health facilities - Ensuring network security, promptly correct 'fake' news.
	• On 8 February, MOH officially launched website https://ncov.moh.gov.vn/ and app named ‘Vietnam Health’ to provide information on nCoV infection [75].
	• On 9 March, the Ministry of Information and Communications and MOH launched 2 apps: 'NCOVI' application for Vietnamese people and 'the Vietnam Health Declaration' application for all visitors entering Vietnam. These two applications provide information to trace suspected cases with COVID-19. Based on the data collected from these applications, the health care system could ensure the fastest and most effective medical assistance as soon as possible. In addition, this is also an official channel for competent state agencies to send recommendations on disease prevention to users [76].
	• On 18 March, the community monitoring system using GPS to monitor the epidemic was activated in Hanoi (Hanoi SMart City) [77].
	• On 24 March, MOH issued Official Dispatch No. 1519/BYT-MT providing guidance on wearing facial masks for citizens and communities [78].
	• On 18 April, the Ministry of Information and Communications launched the 'Bluezone' application. This application can help users to determine whether they had a close contact with COVID-19 patients [64].
	• Additionally, MOH cooperated with WHO to develop infographics on questions and answers regarding nCoV infection prevention; MOH created infographics on recommendations of COVID-19 preventive measures for specific subjects such as drivers, passengers on public transportations..[79].
Economic supports	• On 7 February, the Ministry of Finance announced a list of medical supplies including face masks, hand sanitizers, protective suits and others that are exempted from tax until the epidemic ends [80].
	• On 3 March, the General Department of Taxation issued Official Letter No. 897/TCT-QLN on extension of tax payment, exemption of late payment due to COVID-19 epidemic [81].
	• On 4 March, the Directive No. 11/CT-TTg 2020 on urgent objectives and solutions for assisting businesses facing difficulties and assurance of social welfare amid COVID-19 pandemic issued by the Prime Minister, in which a credit package of VND 250 billion was proposed to support productions and businesses [82].
	• On 20 March, Vietnam’s Ministry of Industry and Trade has decided to cut electricity tariffs by 10 percent for three months [83].
	• On 7 April, Hanoi People's Committee issued Directive No. 06/CT-UBND on reduction of land rent, house rent, reduction of some types of collection fees in Hanoi [84].
	• On 9 April, the government issued Decree No. 41/2020/ND-CP on tax and land rent deferral [85].
	• On 10 April, the government announced plans for a $2.6 billion fiscal package to support people affected by COVID-19 pandemic, issued in Resolution No. 42/ND-CP [86].
	• On 24 April, the Prime Minister issued Decision No. 15/2020/QD-TTg on implementation of policies on assistance for people affected by COVID-19 pandemic [87].
	• People in medical isolation areas including medical centers, concentrated quarantine facilities were given around 80 thousand VND/d in allowance, and all direct medical fees were covered for Vietnamese citizens [88].
Other responses	• On 24 March, Vietnam stopped exporting rice to ensure national food security [89].
	• In March, Hue hospital invented robots to assist serving patients in quarantine areas [90].
	• Additionally, penalties of violations of COVID-19 prevention and control policies were imposed including not wearing masks in public places, concealing health status, opening non-essential businesses, gathering of more than 10 people, spreading ‘fake news’ and others [91].
	• In March and April, a series of meetings among national senior officers were held to discuss health information and updates, as well as to share experiences on COVID-19 prevention, screening, and treatment across ASEAN countries [92].
	• Vietnam transported 450 000 protective suits and 200 000 face masks to the United States, provided medical supplies valued US$ 100 000 to Japan. In addition, 550 000 face masks were donated to European countries and 730 000 to neighboring countries [93].

MOH – Ministry of Health

#### Border closure and entry ban

In the first stage, when the outbreak was only localized in China, on 23 January, the Civil Aviation Authority of Vietnam announced instruction No. 358/CT-CHK regarding a temporary ban of all citizens from Wuhan city, the initial epicenter [7]. The ban was later extended to all flights to and from mainland China [9]. Having a bustling border and a high volume of trade with China, Vietnam was expected to be heavily affected due to the disease’s fast-spreading nature. Recognizing the potential high risks of the disease and limited existing resources, the government focused mainly on preventive measures. Stringent inspections were strictly implemented at airports and land border checkpoints in the form of temperature checks, medical declarations and health checks [20]. All cases with suspicious symptoms were taken in for further diagnosis.

On 12 February, Vietnam and China governments agreed to ease the cross-border restriction to normalize trade in Northern provinces: Quang Ninh, Lai Chau, Lao Cai, Lang Son, and Ha Giang [9]. These trade policy changes had to be carefully considered by the government to strive for the ideal balance between economic and public health security. Trading activities put firm pressure on health workers in the prevention and treatment of COVID-19 in the subsequent disease stages in Vietnam.

As the pandemic widely spread in Deagu, South Korea, the second hotspot after China, the entry ban was further applied to travelers coming from severely affected regions in South Korea, in addition to a mandatory 14-day isolation for all other incoming Korean travelers effective as of 25 February [10]. On 05 March, the Civil Aviation Authority decided to temporarily suspend all flights to South Korea [12]. In this period, a series of emergency and successive restrictions were carried out based on the complex evolution of the disease worldwide, particularly the outbreak in European countries [13]. All international flights were temporarily suspended beginning 19 March and there were considerable reductions in the number of domestic flights [14,43]. Besides, all visa issuance were suspended, except for those on official or diplomatic missions. The border closure timeline in Vietnam was quite aligned with that of other countries in the Southeast Asian region, for example Thailand (22 March), Indonesia (31 March), Singapore (22 March) [3]. It is clear that controlling the epidemic has become the top priority for countries all over the world.

#### Quarantine and lockdown

As of February 2, Prime Minister Nguyen Xuan Phuc enacted the Official Dispatch No. 156/CD-TTg enforcing 14-day quarantine for Vietnamese citizens returning from China [16]. On 3 February, the policy was extended to all citizens who entered Vietnam from 31 provinces of China, including foreigners [17]. In the first stage, the policy largely focused on preventing the penetration from the epicenter Wuhan and China in general.

In the second stage being aware of the pandemic danger, the government strictly implemented quarantine measures. On 1 March, citizens who entered the country from epidemic countries (China, Korea, Italy, Iran) have to fill in a health declaration form and follow medical quarantine [20,21]. The newly confirmed cases were mainly foreigners and Vietnamese citizens who returned from European countries or had direct contact with positive patients. Therefore, isolation and quarantine precautions were taken to curb the rise of disease transmission. Several localities at potential high risks of community transmission in Son Loi-Vinh Phuc, Truc Bach-Hanoi and some communities in Hai Phong, Ha Loi-Me Linh were placed in lockdown [25-28]. Bach Mai, a leading hospital at central level with more than 5000 health care workers was locked down in late March after one staff was confirmed positive for COVID-19 [29].

A 15-day nationwide social distancing period began on 1 April led by the principle that “every province and city going into self-isolation” under the Directive No. 16/CT-TTg. Accordingly, everyone – with the exception of essential workers – was required to “shelter in place” and not go out unless for bare necessities such as buying food or drugs [23]. Although there were several considerable inconveniences in implementation of social distancing, most Vietnamese citizens have complied with the government’s regulation. Whereas in the Philippines, the use of weapons had to be deployed to force habitants to follow the government’s measures. On 4 April, an unfortunate incident was recorded in the Philippines as one protester who refused to follow the restriction orders was shot by the police [3]. Back in Vietnam, the number of confirmed cases during this period slightly increased, with over 250 cases in early April [2].

On 16 April, following 15 days of national social distancing, the government divided all localities of the country into ‘high-risk’, ‘at-risk’, and ‘low-risk’ zones to impose precautions accordingly. Hanoi, Ho Chi Minh city – the two biggest metropolitan cities and 10 other provinces had been implementing strict social distancing measures at least until 22. Starting 23 April, most cities and provinces began loosening social distancing requirements to allow more daily routines while still having protective measures in place. Under the guideline, Hanoi and Ho Chi Minh city were expected to intermittently operate daily services [30]. Meanwhile in Thailand, the Prime Minister announced plans to gradually relax restrictions by the end of April owing to the reduction of new confirmed cases. However, in other countries such as Singapore, Philippines and Indonesia, the strict measures continued to be applied until there were clear signs of case decline [3]. The 15-day period had been considered a significant milestone in the prevention of disease transmission in Vietnam. Although there might have been some undiagnosed cases, it cannot be denied that Vietnam’s approach has been well-organized and effectively reduced the speed of transmission.

#### Shutdown non-essential business, school or workplace closure, suspended transportation

In the first stage of the pandemic on 2 February, the Ministry of Education and Training announced the closure of all public and private educational institutions across the country [35]. During this period, even though the outbreak was mainly confined to China, the prompt decision of school closing and banning public crowds had shown the advantages of limiting community transmission, which is in line with many other countries’ outcomes [94]. However, the unexpected break has caused students and teachers difficulties in the learning and teaching process. In the meantime, the Ministry of Education and Training was quick to launch innovative distant-learning programs on national television to support students in preparing for their entrance exams [95,96].

During the second stage, restriction measures regarding public gathering were tightened. A series of government documents issued in mid-March had highlighted the importance of social distancing measures and Vietnam’s coping strategies reached a climax of highest stringency levels when the 15-day national social distancing measures took effect on 1 April [23]. The prime objective of the measures was to urgently slow the number of infections or to “flatten the curve” of infection. One of the main priorities was to reduce and cease all non-essential business services, including restaurants, gyms, movie theaters, and religious, sports and cultural gatherings [38]. Many companies and agencies had made the rational decision to allow their employees to work from home, which not only reduced the risk of disease transmission, but also maintained the income for employed individuals. In addition, buses and railroad services were suspended both within a province and between provinces [40].

Upon entering a new phase with fewer new cases as of 23 April, the Ministry of Transport allowed the reoperation of domestic flights as well as reopening of essential transport activities in the whole country [44,97]. In addition, since April 23 most schools were ready to welcome students to return after the long lockdown which had been prolonged since the Lunar New Year [48]. To make schools a safe environment to learn after an extended period of closure required much considerations from localities, which had different operating policies depending on each own economic and social context. At the domestic level, these efforts on limiting exposure dramatically showed positive impacts during a persistent process against the pandemic.

#### Improving capacities of health care system

The effective results of the epidemic control to date have been largely contributed by the MOH and their drastic efforts in delivering rapid and decisive responses on all fronts from research, prevention, screening to diagnosis and treatment of COVID-19. In the early days of the outbreak in China, the guideline for diagnosis and treatment of coronavirus infection for Vietnam was already issued by MOH on 16 January and has been continuously updated throughout the epidemic [49-51]. When the COVID-19 outbreak hit Vietnam on 30 Jan, the National Steering Committee and 45 Rapid Response Teams for outbreak prevention and control were established [53,54]. In the wake of the outbreak, MOH’s hotline numbers 19009095 and 19003228 were set up on 1 Feb to address enquiries and concerns, in addition to the local hotlines for counseling provided by each province/city that were launched on 22 March [55,56].

Furthermore, Vietnam has gained substantial achievements in scientific research and development relating to COVID-19. On 7 February, Vietnam became one of the few countries that successfully cultured and isolated the novel coronavirus strain in the laboratory, which would be the premise for further research and development of vaccines, as well as effective prevention interventions against COVID-19 [57]. Moreover, Vietnam officially manufactured the virus detection test kits (RT-PCR and real-time RT-PCR) on 05 March, and was able to manufacture 10 000 test kits per day [60]. To date, the test kit has been approved by WHO and exported to many countries such as: Sweden, Ukraine, Finland, Germany, Italy, Cambodia [98]. The test kit plays a decisive role in the early diagnosis and timely treatment of COVID-19, alongside contact tracing and mass screening in the communities.

Furthermore, a series of guidelines and recommendations on the prevention and control of COVID-19 were released; for example, the guidelines on self-isolation and concentrated quarantine at medical facilities and hotels [65,66,99,100]; guidelines on the prevention and management of COVID-19 for specific vulnerable groups: infants and pregnant women; the elderly and people with chronic diseases [68,69]; recommendations on the prevention of COVID-19 infections in specific places such as apartment buildings, workplaces, public transports, shopping malls, restaurants and others [70-72].

Moreover, the online-based system for medical examination and treatment of COVID-19 infections were launched initially by the Agency of Health Examination and Treatment on 5 February. It was then, officially launched on 18 April by MOH to support hospitals in remote areas regarding medical counselling, consultation, imaging diagnosis, pathology among others [59,64]. The initiative has helped to eliminate geographical and social barriers, and enhanced diagnosis and treatment capacity for COVID-19 in all health facilities.

Furthermore, the government facilitated a robust health care system through issuing a number of Decisions requiring rapid production of medical equipment and suspending exports of anti-COVID-19 drugs to ensure health system capacities during the complicated development of the pandemic [62,63].

#### Information campaigns

Importantly, the government has executed a series of information and communication campaigns to keep the public updated on the most transparent and latest development of the epidemic. On 30 January, communication campaigns via radio, television, newspaper for the prevention and control of the pandemic were launched in all provinces/cities via cooperation between the Department of Health and Department of Information and Communications [73]. On 2 February, the Ministry of Information and Communications issued Directive No. 5/CT-BTTTT strengthening the information campaigns for the prevention and control of the new coronavirus outbreak [74]. Therein, diverse communication channels were set up to reach the entire population. The campaigns consisted of sending text messages to mobile users; producing music videos and short films to widely disseminate anti-COVID information via mass media channels and social networks such as Facebook, Zalo, Youtube, Lotus and trending the hashtag #ICT_anti_nCoV for raising awareness of the epidemic. Through these different streams of media, promotion of awareness and behavioral changes such as wearing masks in public areas and washing hands, was especially timely during the outbreak. Vietnam’s creative communication approach has gone viral and received much attention by viewers, including those from other countries. For example, the parody song 'Ghen Cô Vy', which highlighted the importance of handwashing and personal hygiene to prevent coronavirus infection has gone viral all around the world and was broadcasted on American and French television channels [101].

In line with these activities, websites and applications were sequentially launched such as ‘Vietnam Health’, ‘NCOVI’ ‘Vietnam Health Declaration’, ‘Hanoi Smart City’ and ‘Bluezone’ with the aim of widely disseminating the most updated findings, information, correct misinformation and supporting health care workers to promptly detect suspected cases [64,75-77]. Furthermore, Q&A infographics and leaflets/posters on nCoV infection prevention, which largely targeted drivers and passengers taking public transports, were created and distributed widely [79]. Furthermore, guidance on the correct use of face masks in public was published nationally by MOH on 24 March [78].

#### Economic support

In response to the adverse socioeconomic impacts on businesses, society and individuals created by the epidemic, preferential economic policies and relief measures have been implemented [11]. On 7 February, the Ministry of Finance announced a list of medical supplies exempted from tax until the epidemic ended including face masks, hand sanitizers, protective suits [80]. Subsequently, tax payment in some cases had been exempted or deferred and electricity tariffs have been reduced for three months for individuals and enterprises affected by the epidemic [81,83,85].

The COVID-19 pandemic obviously has caused a stir for ASEAN economies [102]. On par with other countries, Vietnam launched several packages of economic stimulus to revive the economy. The Prime Minister issued Directive No. 11/CP on urgent objectives and solutions for assisting businesses facing difficulties and assurance of social welfare amid COVID-19 pandemic on 4 March, in which a credit package of 250 VND billion was debuted [82]. On 10 April, the government announced plans for an economic stimulus package valued 60.9 trillion VND to assist people, numerous enterprises, household businesses and cooperatives facing difficulties due to the pandemic [86], which was officially implemented by the Prime Minister’s Decision on 24 April [87].

Isolated people in health care centers and concentrated quarantine facilities are entitled to around 80 000 VND in allowance per person per day (US $3.4) and all direct medical costs are covered for Vietnamese citizens [88].

#### Other responses

Since 24 March, Vietnam has stopped exporting rice to ensure national food security due to the outbreak [89]. Applying the latest digital technology, the Hue Central Hospital created robots that assisted health workers in serving isolated patients in quarantine areas [90]. To reinforce social distancing measures, penalties were imposed for noncompliance of measures including not wearing masks in public places, concealment or dishonesty in self-reporting of health, opening of non-essential businesses, gathering of more than 10 people and spreading ‘fake news’ about the COVID-19 outbreak [91].

Alongside the individual country attempts, ASEAN countries were also united in the fight against the COVID-19 pandemic. Meetings among national senior officers were held in March and April to discuss and share country experiences on COVID-19 prevention, screening and treatment [3,92]. Also, enhancement and strengthening of international cooperation on responses to COVID-19 related health threats is one of the crucial points that were voiced by ASEAN nations [3]. The meetings also convened external experts from the United States, South Korea, Japan and China to share state-of-art technical knowledge and to provide financial support to countries for the current and subsequent waves of the COVID-19 outbreak. [3,92].

Vietnam also made strong efforts to coordinate with international communities in the fight against COVID-19 with the spirit of solidarity – “there is no country alone in the fight against the pandemic”. Since the beginning of April, Vietnam has been transferring medical supply aids to regional neighbors including Cambodia, Indonesia, Laos as well as other developed nations such as the United States, Japan and European countries [93].

### Comparison of governments’ responses to the COVID-19 pandemic among Southeast Asian countries

As of 24 April, the disparity of COVID-19 burden in terms of confirmed cases was observed across nations in Southeast Asia. While several countries reported some few hundreds confirmed cases (19 in Laos, 138 in Brunei, 122 in Cambodia, 139 in Myanmar, 270 in Vietnam), other countries suffered more heavily with the cases being 11 178 in Singapore, 5603 in Malaysia, 7775 in Indonesia, 2854 in Thai Lan and 6981 in the Philippines [1]. To curb the number of confirmed cases, Vietnam has issued a vast array of action policies, with the highest stringency level among Southeast Asian countries (Figure S1 in the **Online Supplementary Document**). Particularly, Vietnam was evaluated as the top country ready to exit lockdown, which was based on the 4 out of 6 WHO recommendations in ‘lockdown rollback’ measures [103]. Thus, the positive outcomes seen in Vietnam could be attributable to the strict precautions taken by the government in the battle against COVID-19. Nevertheless, national level responses to the COVID-19 pandemic vary greatly among countries, depending on each’s socio-economic background, health care capacity and political system.

## CONCLUSION

Overall, Vietnam’s COVID-19 strategies could be considered an effective model for limited resource settings. It is fruition of the prompt, proactive and decisive steps taken by the government on all fronts including health care system, security force, economic policies, along with creative and effective communication campaigns. Therefore, this preliminary success from Vietnam may serve as a valuable case study for other countries in outbreak response. However, our research is merely a snapshot focusing on the government’s responses during the first three months of the COVID outbreak. The pandemic is far from over and the country is expected to maintain precaution methods in the upcoming months.

## Additional material

Online Supplementary Document

## References

[1] Coronavirus disease (COVID-2019) situation reports. Available: https://www.who.int/emergencies/diseases/novel-coronavirus-2019/situation-reports. Accessed: 20 April 2020.

[2] COVID-19 Dashboard by the Center for Systems Science and Engineering at John Hopkins UniversityAvailable: https://gisanddata.maps.arcgis.com/apps/opsdashboard/index.html#/bda7594740fd40299423467b48e9ecf6. Accessed: 20 April 2020.

[3] Center for Strategic & International Studies. Southeast Asia Covid-19 Tracker. 2020. Available: https://www.csis.org/programs/southeast-asia-program/southeast-asia-covid-19-tracker-0. Accessed: 26 April 2020.

[4] Vietnam Ministry of Health. Dashboard for COVID-19 statistics Available: https://ncov.moh.gov.vn/. Accessed: 26 April 2020.

[5] Vietnam LawsAvailable: https://thuvienphapluat.vn/en/index.aspx. Accessed 26 April 2020.

[6] Hale T. Sam Webster, Anna Petherick, Toby Phillips, and Beatriz Kira. Oxford COVID-19 Government Response Tracker, Blavatnik School of Government. Data use policy: Creative Commons Attribution CC BY standard. 2020. Available: https://www.bsg.ox.ac.uk/research/research-projects/oxford-covid-19-government-response-tracker. Accessed: 26 April 2020.

[7] The Prime Minister. Directive No.358/CT-CHK - Prevention of acute respiratory infections caused by new strains of Corona virus to aviation activities in Vietnam, 2020.

[8] The Civil Aviation Authority of Vietnam. Directive No.362/CT-CHK - Prevention of acute respiratory infections caused by new strains of Corona virus to aviation activities in Vietnam, 2020.

[9] The Prime Minister. Official Dispatch No. 224/CĐ-TTg on the difficult removal of goods for import, export and transportation across borders. 2020.

[10] The Prime Minister. Directive No.10/CT-TTg on promotion COVID-19 pandemic prevention activities. 2020.

[11] Tran PB Hensing G Wingfield T Atkins A Sidney K Kazibwe J Income security during public health emergencies: the COVID-19 poverty trap in Vietnam. BMJ Glob Health. 2020;5:e002504. 10.1136/bmjgh-2020-002504 32540965 PMC7299029

[12] Vietnam Airlines halted all routes Vietnam - South Korea. Available: https://tuoitre.vn/vietnam-airlines-tam-dung-tat-ca-duong-bay-viet-nam-han-quoc-2020030221433763.htm. Accessed: 20 April 2020.

[13] Vietnam Airlines temporarily flights between Vietnam and France, Malaysia. 2020. Available: https://www.vietnamairlines.com/cn/en/vietnam-airlines/press-room/travel-advisory/2020/0317-EN-VNA-suspend-route-france-malaysia. Accessed: 20 April 2020.

[14] Vietnam Airlines temporarily suspended international flights. 2020. Available: https://www.vietnamairlines.com/vn/en/vietnam-airlines/press-room/travel-advisory/2020/0319-EN-VNA-suspend-international-route. Accessed: 20 April 2020.

[15] Vietnam suspends border crossing from and to Laos, Cambodia. Available: https://vietnaminsider.vn/vietnam-suspends-border-crossing-from-and-to-laos-cambodia/. Accessed: 20 April 2020.

[16] The Prime Minister. Official Telegraph No.156/CĐ-TTg on strengthening prevention and control of acute respiratory infections caused by new strains of corona virus. 2020.

[17] Isolating all people from 31 outbreak provinces, China. Available: https://tuoitre.vn/cach-ly-tat-ca-nhung-nguoi-den-tu-31-tinh-co-dich-cua-trung-quoc-2020020319365586.htm. Accessed: 20 April 2020.

[18] Compulsory isolation citizens from the epidemic areas in Korea. 2020. Available: https://thanhnien.vn/thoi-su/cach-ly-bat-buoc-nguoi-ve-tu-vung-dich-han-quoc-1187572.html. Accessed: 20 April 2020.

[19] Ministry of Transportation. Document No.1637/BGTVT-VT on promptly implement the Prime Minister's Directive No. 10 / CT-TTg on promoting the prevention and control of COVID-19 epidemic. 2020.

[20] Ministry of Transportation & Civil Aviation Authority of Vietnam. Mandatory medical declaration for passengers entering Vietnam. Available:http://img2.caa.gov.vn/2020/03/06/19/13/939CHKQLC.PDF. Accessed: 20 April 2020.

[21] Medical declarations compel all passengers to enter Vietnam. Available: https://moh.gov.vn/hoat-dong-cua-dia-phuong/-/asset_publisher/gHbla8vOQDuS/content/khai-bao-y-te-bat-buoc-moi-hanh-khach-nhap-canh-vao-viet-nam. Accessed: 20 April 2020.

[22] Since 0h 21 March, all people who enter Vietnam must be isolated. 2020. Available: https://tuoitre.vn/tu-0h-ngay-21-3-nguoi-nhap-canh-vao-viet-nam-deu-phai-cach-ly-20200320171258515.htm. Accessed: 20 April 2020.

[23] The Prime Minister. Directive No.16/CT-TTg - Implementation of social distancing nationwide, 2020.

[24] Samsung workers infected with Covid-19, Bac Ninh isolated 44 close contacts cases. 2020. Available: https://thanhnien.vn/thoi-su/cong-nhan-samsung-nhiem-covid-19-bac-ninh-cach-ly-44-truong-hop-tiep-xuc-gan-1210319.html. Accessed: 25 April 2020.

[25] Vinh Phuc Provincial People's Committees. Decision No.269/QĐ-UBND on implementation of emergency responsibilities in Decision no. 173 / QĐ-TTg of 1 February 2020 of the Prime Minister on announcing the emergency situation affected by a new chain virus in Vinh Phuc province 2020.

[26] Hanoi: Eligible to end isolation of Truc Bach street. Available: http://www.hanoimoi.com.vn/tin-tuc/Xa-hoi/961754/ha-noi-du-dieu-kien-ket-thuc-cach-ly–pho-truc-bach. Accessed: 20 April 2020.

[27] Set up 9 medical isolation posts for Ha Loi village, Me Linh commune in 28 days. 2020. Available: https://hanoimoi.com.vn/tin-tuc/Xa-hoi/963737/lap-9-chot-cach-ly-y-te-thon-ha-loi-xa-me-linh-trong-28-ngay. Accessed: 20 April 2020.

[28] Hai Phong isolates six localities as novel coronavirus inflicts mayhem. 2020. Available: https://e.vnexpress.net/news/news/hai-phong-isolates-six-localities-as-novel-coronavirus-inflicts-mayhem-4067048.html. Accessed: 20 April 2020.

[29] Bach Mai Hospital will no longer be isolated from 0h on April 12, and will resume operation in early May. 2020. Available: https://tuoitre.vn/benh-vien-bach-mai-het-cach-ly-tu-0h-ngay-12-4-se-hoat-dong-lai-vao-dau-thang-5-20200412001819298.htm. Accessed: 20 April 2020.

[30] The Prime Minister. Notice No.158/TB-VPCP The plan for social distancing from 16 April across the country, 2020.

[31] The Prime Minister. The Directive No.19/CT-TTg on continuing to implement COVID-19 epidemic control measures in the new situation. 2020.

[32] Hanoi allows school closure until the end of 9 Febuary. 2020. Available: https://tuoitre.vn/ha-noi-cho-hoc-sinh-nghi-hoc-den-het-9-2-20200202195100563.htm. Accessed: 20 April 2020.

[33] Ho Chi Minh City allows students are absent from school for a week to prevent Corona. 2020. Available: https://tuoitre.vn/hoc-sinh-tp-hcm-duoc-nghi-hoc-1-tuan-de-phong-corona-20200202175017186.htm. Accessed: 20 April 2020.

[34] The Prime Minister. The Official Dispatch No.156/CĐ-TTg on 2 February 2020 on strengthening prevention and control of acute respiratory infections caused by new stains of Corona virus. 2020.

[35] The Ministry of Education and Training allows school closure to prevent and fight against nCoV disease. 2020. Available: https://moet.gov.vn/tintuc/Pages/phong-chong-nCoV.aspx?ItemID=6456. Accessed: 20 April 2020.

[36] Ministry of Culture Sport, and Tourism. The Official Telegraph No. 396 / CD-BVHTTDL on strengthening the preventive activities of COVID-19 in communication activities, cultural and historical places. 2020.

[37] The railway industry suspended many trains from today because of COVID-19. 2020. Available: http://baolangson.vn/xa-hoi/276118-nganh-duong-sat-tam-dung-nhieu-doan-tau-tu-hom-nay-vi-covid-19.html. Accessed: 25 April 2020.

[38] Ho Chi Minh city People's Committee - Food Safety Management Authority of Ho Chi Minh City. No.635/BQLATTP-VP on temporarily stop some non-essential activities. 2020.

[39] The Prime Minister. Directive No.15/CT-TTg on drastically implementing the peak phase of covid prevention. 2020.

[40] Hanoi People’s Committee - Hanoi Transport Corporation LTD. Document No.393/TCT-VTHKCC on plan to adjust bus transport activities during the peak of covid prevention. 2020.

[41] Ministry of Transportation. The Official Dispatch No. 2917 / BGTVT-VT on continuing Directive No.15/CT-TTg. 2020.

[42] The airline has experienced unprecedented difficulties in history. 2020. Available: https://bnews.vn/hang-khong-hung-chiu-nhung-kho-khan-chua-tung-co-trong-lich-su/152376.html. Accessed: 25 April 2020.

[43] Ministry of Transportation. Document No.3064/BGTVT-VT on Implementing Directive No. 16 / CT-TTg dated March 31, 2020 of the Prime Minister. 2020.

[44] Ministry of Transportation. Official Dispatch No.3864/BGTVT-VT on implementing the Prime Minister's direction at the Government's Standing Committee on Prevention and Combat of Covid-19 on April 22, 2020.

[45] Ministry of Transportation. Official Dispatch No. 3655/BGTVT-VT on implementation of Announcement No.158/TB-VPCP dated 16/04/2020 of the Prime Minister. 2020.

[46] Provinces and cities wait for the next instruction on social spacing. 2020. Available: https://tuoitre.vn/cac-tinh-thanh-cho-chi-thi-tiep-theo-ve-gian-cach-xa-hoi-20200422081107035.htm. Accessed: 25 April 2020.

[47] Airlines announced simultaneously deploying domestic flights from 16/4. 2020. Available: http://thoibaotaichinhvietnam.vn/pages/kinh-doanh/2020-04-11/cac-hang-hang-khong-thong-bao-dong-loat-trien-khai-bay-noi-dia-tro-lai-tu-ngay-16-4-85252.aspx. Accessed: 25 April 2020.

[48] Update: Return school schedule for students from 63 provinces and cities. 2020. Available: https://thoidai.com.vn/cap-nhat-lich-di-hoc-tro-lai-cua-hoc-sinh-63-tinh-thanh-pho-106645.html. Accessed: 26 April 2020.

[49] Ministry of Health. Decision No.125/QĐ-BYT on guideline for diagnosis and treatment for novel coronavirus infection. 2020.

[50] Ministry of Health. Decision No.322/QĐ-BYT on guideline for diagnosis and treatment for novel coronavirus infection (2019-nCoV). 2020.

[51] Ministry of Health. Decision No.1344/QĐ-BYT on guideline for diagnosis and treatment for SARS-COV-2 (COVID-19). 2020.

[52] The Prime Minister. Directive No. 05 / CT-TTg on prevention and control of Coronavirus outbreak. 2020.

[53] Ministry of Health. Decision No. 255 /QD-BYT on the establishment of a Rapid Response Team to control the novel coronavirus disease (NCOV). 2020.

[54] The Prime Minister. Decision No.170/QD-TTg on establishment National Steering Committee for control the novel coronavirus disease. 2020.

[55] Free calls for Q&A about novel coronavirus disease. Available: https://tuoitre.vn/mien-phi-cac-cuoc-goi-giai-dap-thong-tin-ve-dich-benh-corona-20200201224351027.htm. Accessed: 20 April 2020.

[56] COVID-19 hotline of 63 provinces. Available: https://tuoitre.vn/duong-day-nong-tu-van-covid-19-cua-63-tinh-thanh-20200322135510052.htm. Accessed: 20 April 2020.

[57] Vietnam successfully cultured and isolated new coronavirus strain in laboratory. 2020. Available: https://thanhnien.vn/thoi-su/viet-nam-phan-lap-duoc-virus-corona-gay-dich-viem-duong-ho-hap-cap-1179845.html. Accessed: 20 April 2020.

[58] The National Steering Committee for prevention and control the novel coronavirus disease. Decision No. 80 / QD-BCDQG on establishment Sub-committees to respond the novel coronavirus disease. 2020.

[59] Launching the online-based management and administration center to support COVID-19 diagnosis and treatment. Available: https://thanhnien.vn/suc-khoe/ra-mat-trung-tam-quan-ly-dieu-hanh-ho-tro-chuyen-mon-chan-doan-dieu-tri-covid-19-1191516.html. Accessed: 25 April 2020.

[60] Vietnam officially produced the Sars-Cov-2 detection test kit with a capacity of 10,000 sets/day. 2020. Available: https://thanhnien.vn/thoi-su/viet-nam-che-tao-bo-kit-phat-hien-sars-cov-2-cong-suat-10000-bongay-1191346.html. Accessed: 20 April 2020.

[61] The Ministry of Health imported 200,000 nCoV rapid tests. 2020. Available: https://vnexpress.net/bo-y-te-nhap-200-000-test-xet-nghiem-nhanh-ncov-4076810.html. Accessed: 20 April 2020.

[62] The Government Office. Announcement No. 143/TB-VPCP about the decision at the regular meeting on prevention and control the COVID-19 epidemic. 2020.

[63] The Drug Administration of Vietnam. Official Dispatch No. 4162/QLD-KD stopping anti-COVID-19 drugs exports. Available: https://dav.gov.vn/cong-van-so-4162qld-kd-ve-viec-tam-dung-xuat-khau-thuoc-phong-chong-covid-19-n2826.html. Accessed: 25 April 2020.

[64] Ministry of Health Portal. Prime Minister attended the opening of 2 technology products for prevention of COVID-19. Available: https://moh.gov.vn/hoat-dong-cua-lanh-dao-bo/-/asset_publisher/TW6LTp1ZtwaN/content/thu-tuong-du-khai-truong-2-san-pham-cong-nghe-giup-phong-chong-covid-19. Accessed: 25 April 2020.

[65] Ministry of Health. Decision No. 879/QD-BYT on interim guidance for the COVID-19 quarantine at home and in residential areas. 2020.

[66] Ministry of Health. Decision No.344/QĐ-BYT on guidance on quarantine at quarantine zones for prevention and control of novel coronavirus pneumonia (COVID-19). 2020.

[67] Ministry of Health. Decision No. 904/QD-BYT on the manual for implementation of quarantine at quarantine zones. 2020.

[68] Ministry of Health. Decision No.1271/QD-BYT on interim guidance on prevention and management of SARS-CoV-2 infection (COVID-19) for pregnant women and infants. 2020.

[69] Ministry of Health. Decision No. 1588 / QD-BYT on interim guidance on the health management for the elderly and people with chronic diseases at healthcare facilities in the context of the Covid-19 epidemic; and guidance on health care and prevention Covid-19 epidemic for the elderly in the communities. 2020.

[70] Health Environment Management Agency. The Official Dispatch No. 1364/BYT-MT on prevention and control the COVID-19 at apartment buildings. 2020.

[71] Health Environment Management Agency. The Official Dispatch No. 490/BYT-MT on prevention and control the COVID-19 at workplaces. 2020.

[72] Health Environment Management Agency. Official Dispatch No. 831/BYT-MT on prevention and control the COVID-19 epidemic in shopping malls, supermarkets, traditional markets, restaurants, hotels, parks and tourist areas. 2020.

[73] Ministry of Health. Official Dispatch No. 369/BYT-TT-KT on strengthening propaganda, advocacy of prevention and control of nCoV. 2020.

[74] Ministry of Information and Communications. Directive No. 5/CT-BTTTT on Prevention and control of COVID-19. 2020.

[75] The Ministry of Health officially launched a website on nCoV. 2020. Available: https://congnghe.tuoitre.vn/bo-y-te-chinh-thuc-ra-mat-trang-tin-ve-dich-benh-virus-corona-20200208181554979.htm. Accessed: 20 April 2020.

[76] Launched medical applications for Vietnamese and foreigners. Available: https://ncov.moh.gov.vn/web/guest/-/ra-mat-ung-dung-tro-giup-y-te-cho-nguoi-viet-nam-va-nguoi-nuoc-ngoai. Accessed: 20 April 2020.

[77] SmartCity application supports Hanoi authorities to monitor suspected people with COVID-19. Available: https://tuoitre.vn/ung-dung-smartcity-ho-tro-co-quan-chuc-nang-ha-noi-giam-sat-nguoi-cach-ly-20200320140019871.htm. Accessed: 20 April 2020.

[78] Ministry of Health. Official Dispatch No. 1519 / BYT-MT on guidance of wearing facial masks for people and communities. 2020.

[79] Infographic on COVID-19 2020 Available: https://vtv.vn/infographic.htm. Accessed: 24 April 2020.

[80] Ministry of Finance. Exempting all kinds of taxes for medical materials against nCoV epidemic. Available: http://baochinhphu.vn/Kinh-te/Bo-Tai-chinh-Mien-thue-cac-loai-vat-tu-y-te-chong-dich-nCoV/387058.vgp. Accessed: 24 April 2020.

[81] The General Department of Taxation. Official Letter No. 897 / TCT-QLN on extension of tax payment, exemption of late payment due to Covid-19 epidemic. 2020.

[82] The Prime Minister. Directive No. 11/CP on urgent objectives and solutions for assisting businesses facing difficulties and assurance of social welfare amid COVID-19 pandemic. 2020.

[83] Ministry of Industry and Trade. Official Dispatch No. 2698/BTC-DTDL on cutting electricity bills. 2020.

[84] Hanoi People's Committee. Directive No.06/CT-UBND on drastically implementing a number of solutions in investment and construction management to improve the efficiency of public investment, stabilize production and business, and ensure social security in Hanoi city during Covid-19 epidemic. 2020.

[85] The Goverment. Decree No.41/2020/ND-CP on tax and land rent deferral. 2020.

[86] The Goverment. Resolution No. 42/ND-CP on assistance for people affected by COVID-19 Pandemic. 2020.

[87] The Prime Minister. Decision No.15/2020/QD-TTg on implementation of policies on assistance for people affected by COVID-19 pandemic. 2020.

[88] How much is supported for people in quarantine zones due to COVID-19? Available: https://thanhnien.vn/thoi-su/nguoi-bi-cach-ly-y-te-do-dich-covid-19-duoc-ho-tro-bao-nhieu-tien-1203623.html. Accessed: 20 April 2020.

[89] Vietnam has stopped exporting rice: it is necessary to ensure national food security. 2020. Available: http://kinhtedothi.vn/viet-nam-tam-dung-xuat-khau-gao-can-thiet-de-bao-dam-an-ninh-luong-thuc-378759.html. Accessed: 20 April 2020.

[90] Robot helps patients with COVID-19 in quarantine zones. 2020. Available: https://thanhnien.vn/gioi-tre/doc-dao-robot-phuc-vu-benh-nhan-cach-ly-trong-mua-dich-covid-19-1199187.html. Accessed: 20 April 2020.

[91] Detailed penalties of 13 violations in COVID-19 prevention and control policies 2020. Available: http://baochinhphu.vn/Hoat-dong-dia-phuong/Chi-tiet-muc-phat-13-hanh-vi-vi-pham-quy-dinh-phong-chong-COVID19-tai-Ha-Noi/392005.vgp. Accessed: 25 April 2020.

[92] Association of Southeast Asian Nations. ASEAN Secretariat News. 2020. Available: https://asean.org/category/news/asean-secretariat-news/. Accessed: 25 April 2020.

[93] Vietnamese face masks radiate to the world. 2020. Available: https://tuoitre.vn/khau-trang-viet-nam-toa-ra-the-gioi-20200410110930194.htm. Accessed: 26 April 2020.

[94] School closures caused by Coronavirus (COVID-19). Available: https://en.unesco.org/covid19/educationresponse. Accessed: 20 April 2020.

[95] Online teaching: Effort and determination from disadvantaged localities. 2020. Available: https://moet.gov.vn/tintuc/Pages/tin-tong-hop.aspx?ItemID=6609. Accessed: 20 April 2020.

[96] Television schedule for students across the country from April 27 to May 2. Available: https://vietnamnet.vn/vn/giao-duc/goc-phu-huynh/lich-hoc-tren-truyen-hinh-cho-hoc-sinh-ca-nuoc-tu-27-4-2-5-636658.html. Accessed: 20 April 2020.

[97] Ministry of Transportation. Official Dispatch No.3863/BGTVT-CYT on guiding the medical conditions to prevent the COVID-19 outbreak in vehicles. 2020.

[98] COVID-19 test kit produced in Vietnam has been approved by WHO. 2020. Available: https://tuoitre.vn/bo-kit-xet-nghiem-covid-19-viet-nam-san-xuat-da-duoc-who-cong-nhan-20200426063419839.htm. Accessed: 20 April 2020.

[99] Ministry of Health. Decision No. 345/QD-BYT on self-quarantine guidelines for prevention and control of COVID-19. 2020.

[100] Ministry of Health. Decision No.878/QĐ-BYT on interim guidance for the COVID-19 quarantine at health facilities. 2020.

[101] Vietnam’s anti-COVID-19 song excites ‘Last Week Tonight’ host, int’l viewers. Available: https://tuoitrenews.vn/news/lifestyle/20200303/vietnams-anticovid19-song-excites-last-week-tonight-host-intl-viewers/53286.html. Accessed: 23 April 2020.

[102] ASEAN Policy Brief. Economic Impact of COVID-19 Outbreak on ASEAN. Available: https://asean.org/storage/2020/04/ASEAN-Policy-Brief-April-2020_FINAL.pdf. Accessed: 25 April 2020.

[103] Hale T, Phillips T, Petherick A, Kira B, Angrist N, Aymar K, et al. Lockdown rollback checklist: Do countries meet WHO recommendations for rolling back lockdown? Available: https://www.bsg.ox.ac.uk/sites/default/files/2020-04/2020-04-lockdown-rollback-checklist-research-note.pdf. Accessed: 26 April 2020.

