# Supplementary material for: Rapid response to the COVID-19 pandemic: Vietnam government’s experience and preliminary success
Source: J Glob Health. 2020 Jul 28;10(2):020502. doi: 10.7189/jogh.10.020502 (PMC7567433; doi:10.7189/jogh.10.020502)

**Supplemental Figure 1:** Comparison of stringency index of COVID-19 response between Vietnam and other Southeast Asian countries, over increasing confirmed cases.

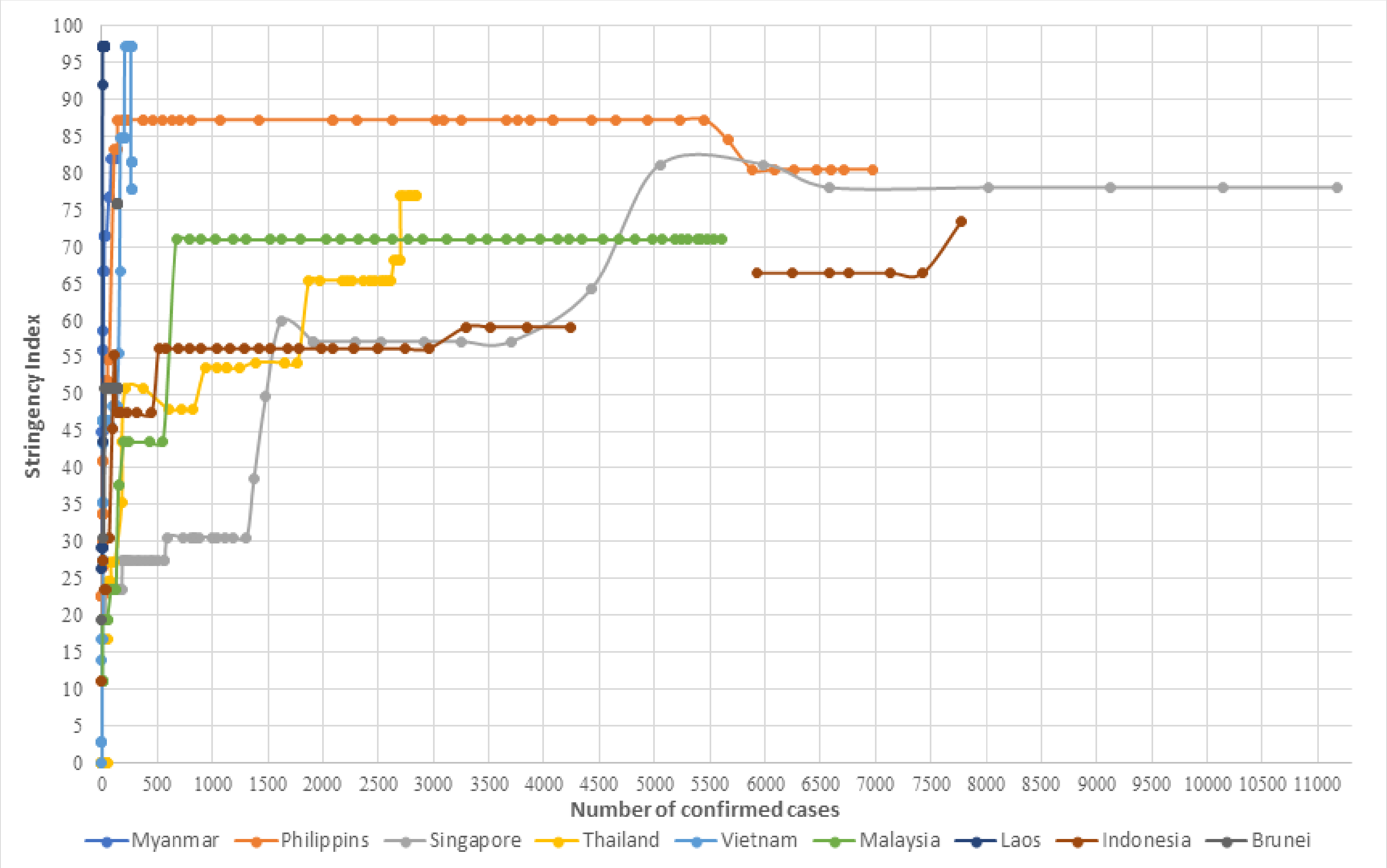

Supplement: Online Supplementary Document [file jogh-10-020502-s001.pdf]
